# Editorial: Fungal secondary metabolites as valuable chemical entities for medicines and agrochemicals

**DOI:** 10.3389/fmicb.2023.1150023

**Published:** 2023-02-06

**Authors:** Weiyi Wang, Ting-Ting Wang

**Affiliations:** ^1^Key Laboratory of Marine Biogenetic Resources, Third Institute of Oceanography, Ministry of Natural Resources, Xiamen, China; ^2^Li Dak Sum Marine Biopharmaceutical Research Center, Department of Marine Pharmacy, College of Food and Pharmaceutical Sciences, Ningbo University, Ningbo, China

**Keywords:** fungal secondary metabolites, biological activities, chemodiversity, polyketides, alkaloids

Natural products, metabolites derived from animals, plants, and microorganisms have a long history as sources of medical and agricultural chemicals. Fungi are a rich source of bioactive natural products. They produce large amounts of compounds with very diverse structural types, such as polyketides, alkaloids, terpenes, and peptides. These metabolites were proven to possess various biological activities, such as antibacterial, antiviral, antitumor, anti-inflammatory, and antiparasitic activities. Some chemicals have already been developed into medicines or pesticides. Examples include penicillin (a β-lactam antibiotic) and lovastatin (a cholesterol-lowering drug). However, the emergence and development of drug resistance in the fields of medicine and agriculture makes it necessary to constantly search for molecules with new mechanisms of action or better activities.

This Research Topic aims to present research progresses and review papers focusing on novel fungal secondary metabolites with high medical and/or agricultural potentials. In this Research Topic, 11 papers have been published: three review papers and eight original research articles.

Some review papers have already discussed secondary metabolites from certain genera of fungi, such as cytotoxic metabolites from *Penicillium* (Koul and Singh, 2017) and bioactive metabolites from marine *Aspergillus* (Wang and Ding, 2018). In this Research Topic, two review papers summarized the secondary metabolites of the *Talaromyces* and the *Alternaria* species. Lei et al. reviewed the chemical constituents of the genus *Talaromyces*, which yield diverse secondary metabolites with various biological activities. Zhao et al. reviewed the products of the genus *Alternaria* focusing mainly on their structural features, various bioactivities, and possible biosynthetic pathways.

In addition to focusing on different genera of fungi, some review articles focused on certain types of metabolites. Diterpenes from marine-derived fungi (Qiu et al., 2022) and alkaloids from endophytic fungi (Daley and Cordell, 2021) are two examples. Chen et al. reviewed the chemodiversity and bioactivities of halometabolites from marine-derived fungi. It was discovered that many brominated and iodinated compounds were generated by the substitution of bromide and iodide ions for the chloride ion during the cultivation process. This confirms the importance of culture conditions on the final products of fungi.

In this Research Topic, we also included some articles reporting the isolation, structure elucidation, and bioactivity evaluation of metabolites derived from different fungi. Three papers were about *Penicillium* strains and two papers were about the genus of *Aspergillus* and *Trichoderma*, respectively. There were reports on different types of metabolites, such as polyketides and alkaloids, with various bioactivities, namely cytotoxic, antimicrobial, anti-inflammatory, and anti-pulmonary fibrosis activities.

Weng et al. found ten metabolites from *Penicillium oxalicum* 2021CDF-3, an endophyte of the marine red algae. The new polyketide oxalihexane A showed a remarkable inhibitory effect on the human pancreatic cancer PATU8988T cell line. The treatment with oxalihexane A down-regulated the expression level of Cyclin D1. Shi et al. isolated seven spirooxindole alkaloids from a terrestrial strain of *Penicillium brefeldianum* and evaluated their antimicrobial activities toward several pathogenic strains. The compound 12α-hydroxyverruculogen TR-2 displayed moderate inhibitory activity toward the dimorphic switch of pathogenic smut fungi *Sporisorium scitamineum*. Weng et al. obtained eight compounds from *Penicillium* sp. YT2019-3321, an endophytic fungus of Lonicera japonica. A new polyketide, penicidone E, showed cytotoxicity against the human pancreatic tumor cells PATU8988T. Xu et al. obtained four indole alkaloids and four polyketides from the deep-sea-derived fungus *Aspergillus flavipes* DS720. The compound flavonoid A showed broad-spectrum cytotoxicities against HeLa, 5637, CAL-62, PATU8988T, A-375, and A-673 cell lines. Hao et al. isolated 25 compounds from the deep-sea fungus *Trichoderma* sp. MCCC 3A01244. The newly identified β-carboline alkaloid trichocarboline A was found to decrease pulmonary fibrosis by inhibiting the TGF-β/Smad signaling pathway.

Although more and more secondary metabolites were obtained from fungi, the exploitation of their biosynthetic potential is far from sufficient. It is believed that by changing the cultivation conditions and growth media composition we can trigger the secondary metabolic pathways. In this Research Topic, Sequeira et al. tried to activate the production of fungal secondary metabolites by supplementing cholinium-based ionic liquids to the growth media of *Neurospora crassa, Aspergillus nidulans*, and *Aspergillus fumigatus*. Both the diversity of metabolites and the levels of certain compounds were increased. Also, the change in bioactivities of the organic extracts was observed. Their work proved that the altering of media components can lead to the changing of fungal products.

In addition to newly discovered compounds, the activity evaluation using different cell lines or models also contributes to the development of leads and drugs, as does the further study of activity mechanisms of the “old” compounds. This Research Topic included two articles reporting the activity evaluation of certain compounds from the genus *Trichoderma*. Huo et al. evaluated the anti-inflammatory activity of trichodimerol, which was first isolated from *Trichoderma longibraciatum*. Trichodimerol was found to reduce the production of NO, ROS, interleukin (IL)-6, and the tumor necrosis factor (TNF)-α. It could also inhibit the production of some inflammatory mediators as well as the expression of some proteins. It was thus concluded that trichodimerol may inhibit inflammation through the NF-κB and NLRP3 pathways. Zhang et al. evaluated the antibacterial effects of TKA, peptaibols produced by *Trichoderma longibrachiatum* SMF2, against the pathogen *Xanthomonas oryzae* pv. *oryzae* (*Xoo*). They found that TKA could significantly inhibit the growth of *Xoo*. The lesion length on the rice leaf was significantly reduced when treated with TKA. Mechanism analyses revealed that TKA treatments resulted in the damage of *Xoo* cell morphology and the release of intracellular substances.

It is expected that this Research Topic will promote interest in the research of agricultural and medical active metabolites derived from fungi.

## Author contributions

WW wrote the manuscript. T-TW revised the manuscript. The final draft of the manuscript was finalized and approved for publication by all authors.
